# Efficacy and safety of risankizumab in Japanese patients with generalized pustular psoriasis or erythrodermic psoriasis: Primary analysis and 180‐week follow‐up results from the phase 3, multicenter IMMspire study

**DOI:** 10.1111/1346-8138.16667

**Published:** 2022-12-13

**Authors:** Keiichi Yamanaka, Yukari Okubo, Ikuko Yasuda, Nobuo Saito, Izabella Messina, Akimichi Morita

**Affiliations:** ^1^ Department of Dermatology Mie University Graduate School of Medicine Tsu Japan; ^2^ Department of Dermatology Tokyo Medical University Tokyo Japan; ^3^ AbbVie GK Tokyo Japan; ^4^ AbbVie Inc. North Chicago Illinois USA; ^5^ Department of Geriatric and Environmental Dermatology Nagoya City University Graduate School of Medical Sciences Nagoya Japan

**Keywords:** erythrodermic psoriasis, generalized pustular psoriasis, interleukin 23, Japanese, psoriasis, risankizumab

## Abstract

Risankizumab, a humanized immunoglobin G1 monoclonal antibody that specifically inhibits interleukin 23 by binding to its p19 subunit, is approved in Japan to treat numerous indications, including generalized pustular psoriasis (GPP) and erythrodermic psoriasis (EP). Both GPP and EP are severe forms of psoriasis that have limited treatment options. In IMMspire (A Study to Assess Efficacy and Safety of Two Different Dose Regimens of Risankizumab Administered Subcutaneously in Japanese Subjects With Generalized Pustular Psoriasis or Erythrodermic Psoriasis) (NCT03022045), a phase 3, randomized, multicenter study in Japan, we evaluated the efficacy and safety of risankizumab for Japanese adults with GPP or EP. Patients were randomized (1:1) to receive open‐label risankizumab 75 mg or 150 mg at weeks 0 and 4 and every 12 weeks thereafter through week 160. The primary efficacy end point was GPP or EP clinical response at week 16. Other efficacy end points included GPP or EP clinical response, ≥90% improvement from baseline in the Psoriasis Area and Severity Index (PASI 90) and Dermatology Life Quality Index of 0 or 1 (DLQI 0/1) through 180 weeks (last follow‐up visit). Safety was assessed throughout. A total of 17 patients (eight with GPP and nine with EP) were enrolled. All patients achieved the primary end point of GPP or EP clinical response at week 16. Among patients continuing risankizumab treatment, achievement of GPP or EP clinical response, PASI 90 and DLQI 0/1 were generally sustained throughout the treatment. The safety profile remained consistent with the safety profiles noted in previous risankizumab studies. Risankizumab demonstrated clinically meaningful efficacy at week 16, with durable efficacy and a favorable long‐term safety profile in Japanese patients with GPP or EP.

## 1 INTRODUCTION

Generalized pustular psoriasis (GPP) and erythrodermic psoriasis (EP) are severe and rare forms of psoriasis. In patients with GPP, numerous tiny, sterile pustules cover the entire body and may cause a fatal multisystem disorder.^1^ GPP may follow an intermittent, relapsing course of flares or attacks that may last for months or years.^2^ In patients with EP, widespread erythema develops across most of the body, and people affected may have a higher risk of developing life‐threatening medical conditions such as hypothermia, hypoalbuminemia, and heart failure.^1^ The prevalence of GPP and EP in Japan are estimated to be 1.1% and 0.4%, respectively.^3^ The rarity of GPP and EP has limited the development of therapy, and current treatment options, including high‐dose corticosteroids, methotrexate, retinoids, antibiotics, and tumor necrosis factor α inhibitors, have variable efficacy and are associated with complications.^4^ Thus, the management of GPP and EP can be challenging as the disease conditions may be severe to potentially life‐threatening and are often resistant to standard therapeutic options.

Risankizumab, a humanized immunoglobin G1 monoclonal antibody that specifically inhibits interleukin 23 by binding to its p19 subunit, is approved in Japan to treat plaque psoriasis, GPP, EP, and psoriatic arthritis in adults.^5^, ^6^, ^7^ To date, no randomized, double‐blind clinical studies have evaluated biologic treatment in patients with GPP and EP because of the limited number of patients,^7^, ^8^ and evidence of the safety and efficacy of risankizumab in patients with GPP or EP is still lacking. Here, we report the primary and long‐term efficacy and safety data of risankizumab in treating Japanese patients with GPP or EP.

## 2 METHODS

### 2.1 Patients

Eligible patients were aged 20 years or older and diagnosed with GPP or EP. Patients with GPP were diagnosed at least 60 days before informed consent was obtained, based on the Japanese Dermatological Association (JDA) criteria, and had an erythema area with pustules accounting for ≥10% of their body surface area and a JDA total severity score < 14. Patients with EP had inflammatory erythema accounting for ≥80% of their body surface area at screening. All patients with GPP or EP were candidates for systemic or phototherapy per the investigator's clinical judgment. Patients who had previous risankizumab exposure, medication‐induced or medication‐exacerbated EP, chronic or relevant acute infections (e.g., HIV, viral hepatitis, active tuberculosis), or other active ongoing inflammatory diseases that could potentially confound trial evaluations were excluded from the study.

### 2.2 Study design and treatment

IMMspire (A Study to Assess Efficacy and Safety of Two Different Dose Regimens of Risankizumab Administered Subcutaneously in Japanese Subjects With Generalized Pustular Psoriasis or Erythrodermic Psoriasis) (NCT03022045) was a phase 3, randomized, open‐label, parallel‐design study that compared two different doses of risankizumab in patients with GPP or EP. After screening, patients were randomized 1:1 to receive subcutaneous administration of open‐label risankizumab 150 mg or 75 mg at week 0 and week 4 and every 12 weeks thereafter through week 160. After marketing approval of risankizumab in Japan, the approved dosage and administration (risankizumab 150 mg at week 0 and week 4 and every 12 weeks thereafter [75 mg may be acceptable for some patients according to their condition]) was followed; no dose adjustments were made after approval. At week 16, patients who failed to achieve clinical response with risankizumab 75 mg were allowed to increase the dose to 150 mg. One follow‐up visit was conducted 20 weeks after the last administration of the study drug. This clinical study was conducted in accordance with the protocol, International Council for Harmonization of Technical Requirements for Pharmaceuticals for Human Use guidelines, and applicable guidelines and regulations governing ethical principles originating in the Declaration of Helsinki. An independent ethics committee/institutional review board ensured the ethical, scientific, and medical appropriateness of the study before it was conducted and approved all relevant documentation.

### 2.3 Assessments

#### 2.3.1 Efficacy

The primary end point was the proportion of patients achieving clinical response at week 16. Clinical response was defined as “slightly improved” in the overall improvement rating from baseline according to the JDA total score for patients with GPP and as at least “minimally improved” in the Clinical Global Impression‐Global Improvement Scale for patients with EP. Secondary end points included the proportion of patients achieving clinical response at week 52 and ≥ 90% improvement in the Psoriasis Area and Severity Index (PASI 90) at weeks 16 and 52. Other efficacy end points assessed through week 180 (the last follow‐up visit) included achievement of clinical responses, PASI 90, Dermatology Life Quality Index of 0 or 1 (no effect) (DLQI 0/1), and change from baseline in total JDA scores (0 [best] to 17 [worst]) for patients with GPP.

#### 2.3.2 Safety

Evaluation of safety included monitoring of adverse events (AEs), serious AEs (SAEs), changes in vital signs and clinical laboratory tests, and local tolerability. Laboratory abnormalities were classified by the National Cancer Institute Common Terminology Criteria for Adverse Events.

### 2.4 Statistical analysis

The sample size was not powered for any hypothesis testing because of the limited number of enrolled patients; thus, the sample size was determined based on feasibility. All efficacy and safety analyses were conducted for the intent‐to‐treat population (all randomized patients who received one or more dose of the study drug). No patients were excluded from the analyses. In addition to the as‐observed analysis, nonresponder imputation and last‐observation‐carried‐forward analyses were used to handle missing data for binary and continuous end points, respectively.

## 3 RESULTS

### 3.1 Patients

**FIGURE 1:**
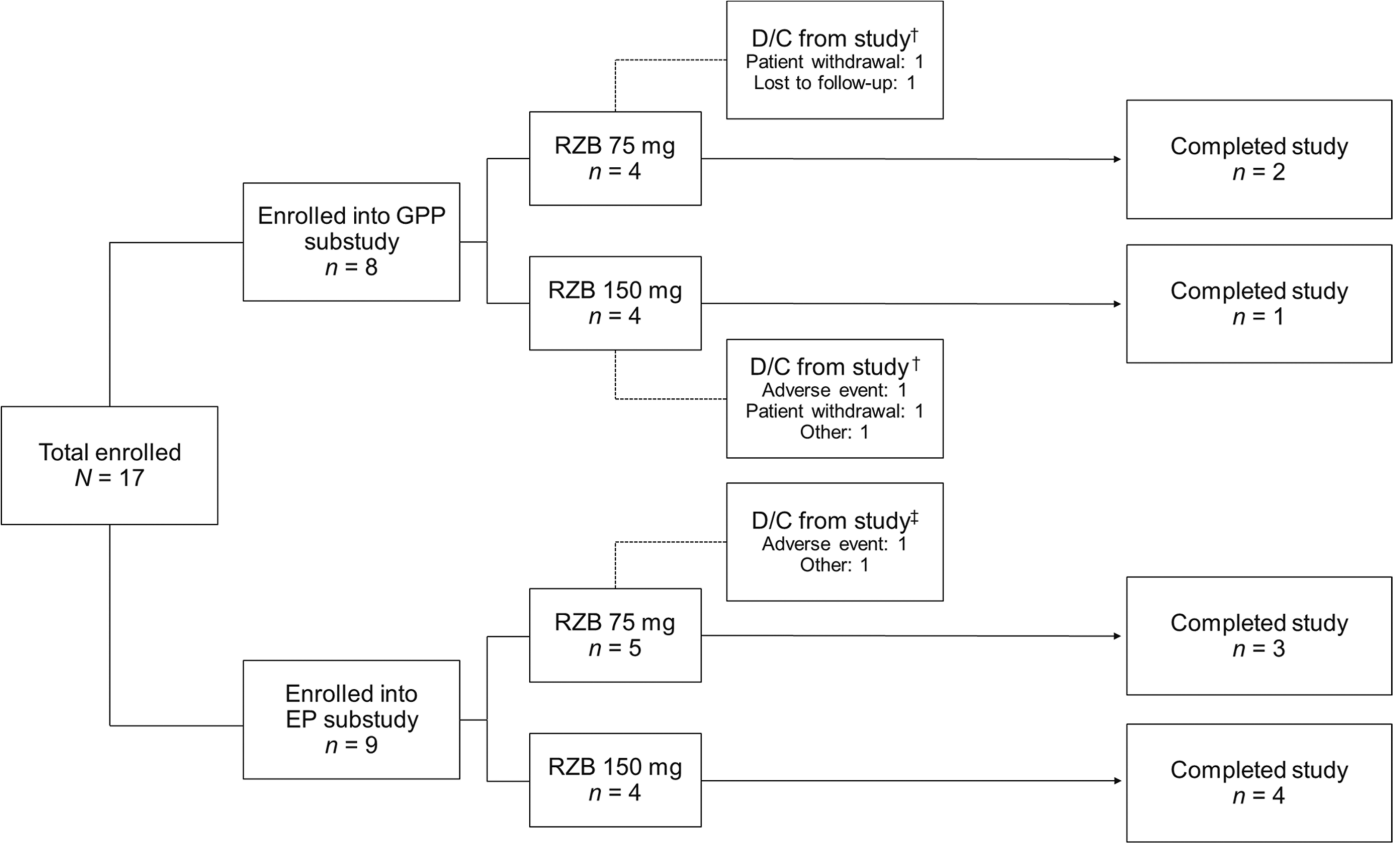
Patient disposition. ^†^There were five study discontinuations in the generalized pustular psoriasis (GPP) group (one due to lost to follow‐up, one due to noncompliance with the protocol, one due to a serious adverse event [AE] of scirrhous gastric cancer that was unrelated to study drug, and two due to withdrawal by patient [one due to need for prohibited medication to treat a nonserious AE of inflammation, pain in extremity, and rash and one due to patient desire to stop tuberculosis prophylaxis medication]). ^‡^There were two study discontinuations in the erythrodermic psoriasis (EP) group (one due to a serious AE of ischemic heart failure that was unrelated to study drug and one due to an unintended pregnancy). D/C, discontinuation; RZB, risankizumab.

A total of 17 patients, including eight patients with GPP and nine patients with EP, were enrolled and 10 (58.8%) patients completed the study **(**Figure 1 **)**. The mean age for patients with GPP was 57.5 years (standard deviation [SD], 18.7 years), and for patients with EP was 40.2 years (SD, 18.8 years); 82.4% were men (Table 1). At baseline, mean PASI was 17.4 (SD, 9.4) for patients with GPP and 52.1 (SD, 13.6) for patients with EP. All patients with GPP had a JDA score of mild (0–6) or moderate (7–10) at baseline; the mean JDA score was 4.8. All patients also had an overlapping comorbidity of psoriatic arthritis. Prior biologic therapy use was reported for three (37.5%) patients with GPP and two (22.2%) patients with EP.

**TABLE 1:** Baseline demographics and disease characteristics

Characteristic	GPP			EP		
	Risankizumab 75 mg (*n* = 4)	Risankizumab 150 mg (*n* = 4)	Total (*n* = 8)	Risankizumab 75 mg (*n* = 5)	Risankizumab 150 mg (*n* = 4)	Total (*n* = 9)
Age, mean (SD), (years)	61.5 (12.5)	53.5 (24.9)	57.5 (18.7)	44.6 (24.2)	34.8 (9.5)	40.2 (18.8)
Men, *n* (%)	4 (100)	2 (50.0)	6 (75.0)	4 (80.0)	4 (100)	8 (88.9)
Weight, mean (SD), (kg)	68.8 (2.9)	59.3 (16.1)	64.1 (11.9)	69.9 (17.4)	89.7 (15.0)	78.7 (18.6)
BMI, mean (SD), (kg/m^2^)	24.2 (1.1)	23.6 (6.3)	23.9 (4.2)	25.0 (6.7)	29.9 (5.0)	27.1 (6.2)
PASI, mean (SD)	11.5 (3.9)	23.3 (10.0)	17.4 (9.4)	46.7 (16.1)	58.7 (6.8)	52.1 (13.6)
BSA, mean (SD), (%)	26.0 (9.9)	36.8 (10.6)	31.4 (11.1)	91.0 (7.7)	94.3 (3.8)	92.4 (6.2)
JDA score						
Baseline, mean	4.5	5.0	4.8	NA^a^	NA^a^	NA^a^
Mild, *n* (%)	4 (100)	1 (25.0)	5 (62.5)	NA^a^	NA^a^	NA^a^
Moderate, *n* (%)	0	3 (75.0)	3 (37.5)	NA^a^	NA^a^	NA^a^
PGA‐GPP, *n* (%)						
Minimal	2 (50.0)	0	2 (25.0)	NA^a^	NA^a^	NA^a^
Moderate	0	2 (50.0)	2 (25.0)	NA^a^	NA^a^	NA^a^
Severe	0	1 (25.0)	1 (12.5)	NA^a^	NA^a^	NA^a^
DLQI 0/1, *n* (%)	2 (50.0)	0	2 (25.0)	0	0	0
Psoriatic arthritis, *n* (%)	4 (100)	4 (100)	8 (100)	5 (100)	4 (100)	9 (100)
Any prior biologic therapy, *n* (%)	2 (50.0)	1 (25.0)	3 (37.5)	1 (20.0)	1 (25.0)	2 (22.2)
TNFi	1 (25.0)	1 (25.0)	2 (25.0)	1 (20.0)	1 (25.0)	2 (22.2)
Non‐TNFi	1 (25.0)	0	1 (12.5)	0	0	0

Abbreviations: BMI, body mass index; BSA, body surface area; DLQI 0/1, Dermatology Life Quality Index of 0 or 1 (no effect); EP, erythrodermic psoriasis; JDA, Japanese Dermatological Association; NA, not applicable; PASI, Psoriasis Area and Severity Index; PGA‐GPP, Physician's Global Assessment of Generalized Pustular Psoriasis; SD, standard deviation; TNFi, tumor necrosis factor α inhibitor.

^a^ Only applicable to patients with generalized pustular psoriasis (GPP).

### 3.2 Efficacy

#### 3.2.1 Patients with GPP

**FIGURE 2:**
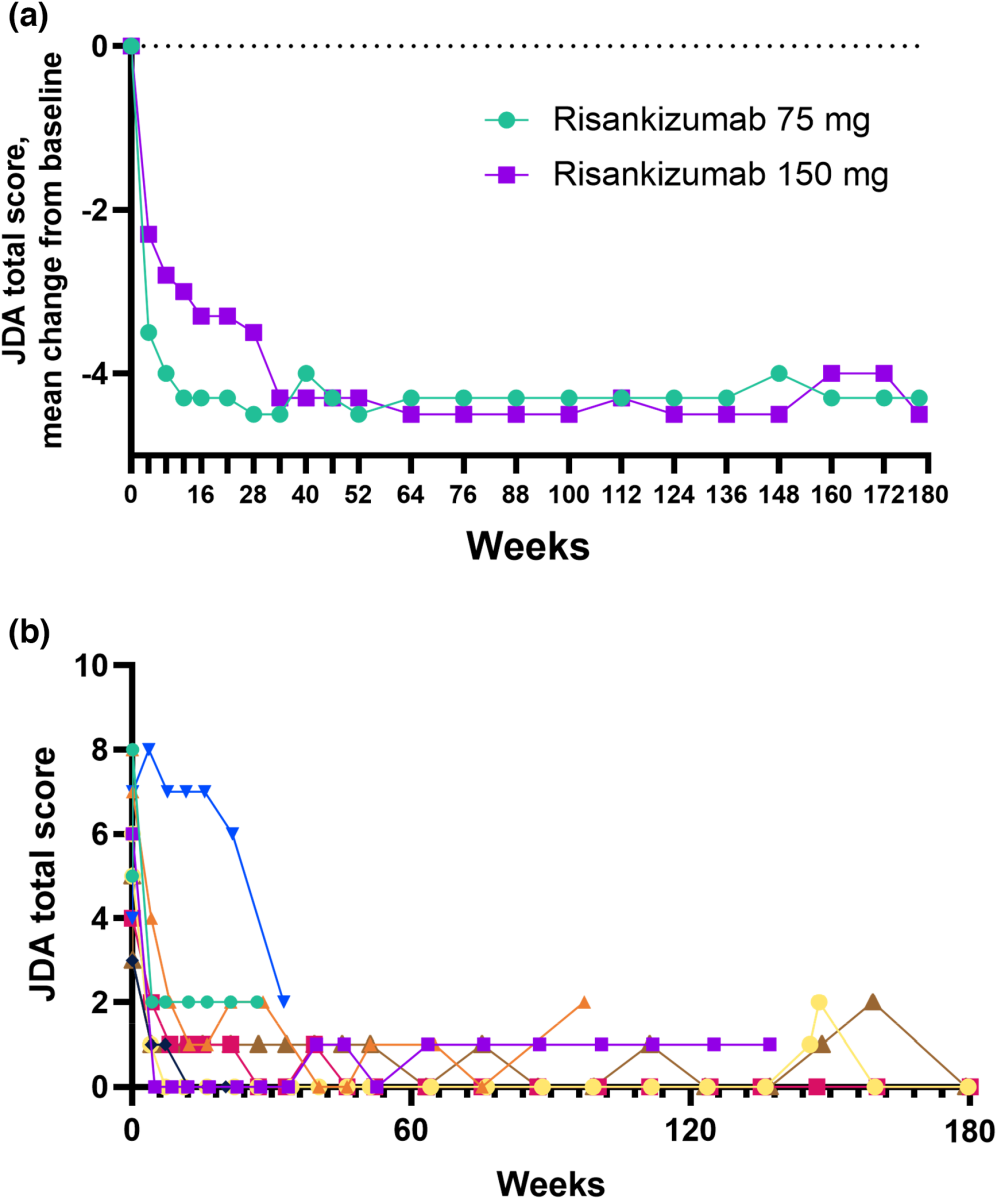
Japanese Dermatological Association (JDA) total score over time in patients with generalized pustular psoriasis (GPP). (a) Mean change from baseline by visit. (b) JDA total score for individual patients; each colored line represents one patient.

All patients with GPP achieved the primary end point of clinical response at week 16 with risankizumab treatment, regardless of dose (Table 2), and all patients who continued in the study maintained clinical response through week 160. Reduction of JDA total scores from baseline was observed as early as week 4 (mean change = −3.5 [SD, 1.9]) and continued to decrease through week 52. After week 52, JDA total scores were generally maintained through week 160 (Figure 2a). A reduction of JDA total scores from baseline was also observed for individual patients through week 160 (Figure 2b). PASI 90 was achieved by 87.5% of patients with GPP at week 16, and most patients who continued in the study maintained PASI 90 through week 160. After 16 weeks of treatment, DLQI 0/1 was achieved by 75.0% of patients. This rate remained stable among patients who continued in the study, and most of the continuing patients achieved DLQI 0/1 by week 160. Efficacy end points were generally maintained from end of treatment (week 160) through the last follow‐up visit (week 180).

**TABLE 2:** Efficacy of risankizumab in Japanese patients with GPP or EP

Parameter	Nonresponder imputation, *n* (%) [95% CI]				Observed cases, *n/n* (%) [95% CI]			
	GPP		EP		GPP^a^		EP^b^	
	75 mg (*n* = 4)	150 mg (*n* = 4)	75 mg (*n* = 5)	150 mg (*n* = 4)	75 mg	150 mg	75 mg	150 mg
Clinical response^c^								
Week 16 (primary)	4 (100)	4 (100)	5 (100)	4 (100)	4/4 (100)	4/4 (100)	5/5 (100)	4/4 (100)
Week 52 (secondary)	3 (75.0) [32.6–100]	2 (50.0) [1.0–99.0]	5 (100)	4 (100)	3/3 (100)	2/2 (100)	5/5 (100)	4/4 (100)
Week 124	3 (75.0) [32.6–100]	1 (25.0) [0–67.4]	3 (60.0) [17.1–100]	4 (100)	3/3 (100)	1/1 (100)	3/3 (100)	4/4 (100)
Week 160	2 (50.0) [1.0–99.0]	1 (25.0) [0–67.4]	3 (60.0) [17.1–100]	4 (100)	2/2 (100)	1/1 (100)	3/3 (100)	4/4 (100)
Week 180 (follow‐up visit)	2 (50.0) [1.0–99.0]	1 (25.0) [0–67.4]	2 (40.0) [0–82.9]	2 (50.0) [1.0–99.0]	2/2 (100)	1/1 (100)	2/2 (100)	2/2 (100)
PASI 90								
Week 16 (primary)	4 (100)	3 (75.0) [32.6–100]	3 (60.0) [17.1–100]	4 (100)	4/4 (100)	3/4 (75.0) [32.6–100]	3/5 (60.0) [17.1–100]	4/4 (100)
Week 52 (secondary)	3 (75.0) [32.6–100]	2 (50.0) [1.0–99.0]	4 (80.0) [44.9–100]	4 (100)	3/3 (100)	2/2 (100)	4/5 (80.0) [44.9–100]	4/4 (100)
Week 124	2 (50.0) [1.0–99.0]	1 (25.0) [0–67.4]	3 (60.0) [17.1–100]	4 (100)	2/3 (66.7) [13.3–100]	1/1 (100)	3/3 (100)	4/4 (100)
Week 160	2 (50.0) [1.0–99.0]	0	3 (60.0) [17.1–100]	4 (100)	2/2 (100)	0/1 (0)	3/3 (100)	4/4 (100)
Week 180 (follow‐up visit)	2 (50.0) [1.0–99.0]	1 (25.0) [0–67.4]	2 (40.0) [0–82.9]	2 (50.0) [1.0–99.0]	2/2 (100)	1/1 (100)	2/2 (100)	2/2 (100)
DLQI 0/1								
Week 16 (primary)	3 (75.0) [32.6–100]	3 (75.0) [32.6–100]	2 (40.0) [0–82.9]	4 (100)	3/4 (75.0) [32.6–100]	3/4 (75.0) [32.6–100]	2/5 (40.0) [0–82.9]	4/4 (100)
Week 52 (secondary)	2 (50.0) [1.0–99.0]	2 (50.0) [1.0–99.0]	4 (80.0) [44.9–100]	4 (100)	2/3 (66.7) [13.3–100]	2/2 (100)	4/5 (80.0) [44.9–100]	4/4 (100)
Week 124	2 (50.0) [1.0–99.0]	1 (25.0) [0–67.4]	2 (40.0) [0–82.9]	4 (100)	2/3 (66.7) [13.3–100]	1/1 (100)	2/3 (66.7) [13.3–100]	4/4 (100)
Week 160	2 (50.0) [1.0–99.0]	0 (0)	3 (60.0) [17.1–100]	4 (100)	2/2 (100)	0/1 (0)	3/3 (100)	4/4 (100)
Week 180 (follow‐up visit)	2 (50.0) [1.0–99.0]	1 (25.0) [0–67.4]	2 (40.0) [0–82.9]	2 (50.0) [1.0–99.0]	2/2 (100)	1/1 (100)	2/2 (100)	2/2 (100)

Abbreviations: CI, confidence interval; DLQI 0/1, Dermatology Life Quality Index of 0 or 1 (no effect); PASI 90; ≥90% reduction from baseline in Psoriasis Area and Severity Index.

^a^ Three patients with generalized pustular psoriasis (GPP) discontinued after week 16, one discontinued after week 52, and one discontinued after week 124.

^b^ Two patients with erythrodermic psoriasis (EP) discontinued after week 52.

^c^ Clinical response is defined as the proportion of patients achieving GPP clinical response (“slightly improved” in the overall improvement rating from baseline according to the Japanese Dermatological Association [JDA] total score) for patients with GPP or the proportion of patients achieving EP clinical response (“minimally improved” in the Clinical Global Impression‐Global Improvement scale) for patients with EP.

#### 3.2.2 Patients with EP

Similarly, all patients with EP achieved the primary end point of clinical response with risankizumab treatment, regardless of dose (Table 2). All patients who continued in the study maintained clinical response through week 160. PASI 90 was achieved by 77.8% of patients with EP at week 16. Among patients who continued in the study, the proportion achieving PASI 90 increased by week 52, and all continuing patients achieved PASI 90 at week 160. After 16 weeks of treatment, DLQI 0/1 was achieved by 66.7% of patients; this percentage increased over time among patients who continued in the study, and all continuing patients achieved DLQI 0/1 at week 160. Efficacy end points were generally maintained from end of treatment (week 160) through the last follow‐up visit (week 180).

#### 3.2.3 Safety

Treatment‐emergent AEs were reported for most (88.2%) patients (Table 3). AEs reported for two patients or more with GPP or EP were nasopharyngitis (10 patients), abnormal hepatic function (two patients), influenza (two patients), dehydration (two patients), and rash (two patients). SAEs were reported for four patients; however, all SAEs were deemed not to be related to study drug. No patients discontinued study drug before the primary efficacy end point at week 16. AEs leading to drug discontinuation after week 16 were reported for three patients (one due to scirrhous gastric cancer; one due to cardiac failure; and one due to nonserious AE of inflammation, pain in extremity, and rash; all AEs were deemed to be unrelated to study drug). Most AEs reported were mild or moderate in severity. Incidences of possible drug‐induced liver injury occurred in four patients. Of these, two patients had AEs that were deemed to have a reasonable possibility of being related to study drug; one patient experienced nonserious events of elevated alanine aminotransferase levels (moderate), aspartate aminotransferase levels (mild), and gamma‐glutamyl transferase levels (mild); one patient experienced an elevated bilirubin level (mild). An adjudicated cardiovascular event was reported in one patient with EP. One patient with EP in the risankizumab 75‐mg group who was a former smoker with a medical history of diabetes and obesity experienced a fatal AE of cardiac failure, which was deemed unrelated to the study drug.

**TABLE 3:** Safety overview of risankizumab in Japanese patients with GPP or EP

TEAEs, *n* (%)	GPP		EP	
	75 mg (*n* = 4)	150 mg (*n* = 4)	75 mg (*n* = 5)	150 mg (*n* = 4)
Any AE	4 (100)	4 (100)	3 (60.0)	4 (100)
Drug‐related AE^a^	3 (75.0)	1 (25.0)	0	2 (50.0)
Serious AE	0	2 (50.0)^b^ ^,^ ^c^	1 (20.0)^d^	1 (25.0)^e^
Drug‐related serious AE^a^	0	0	0	0
AE leading to drug discontinuation	0	2 (50.0)^b^ ^,^ ^f^	1 (20.0)^g^	0
Adjudicated cardiovascular event	0	0	1 (20.0)^g^	0
Possible drug‐induced liver injury	2 (50.0)^h^ ^,^ ^i^	1 (25)^j^	0	1 (25.0)^k^
Death	0	0	1 (20.0)^g^	0

Abbreviations: AE, adverse event; EP, erythrodermic psoriasis; GPP, generalized pustular psoriasis; TEAE, treatment‐emergent adverse event.

^a^ As assessed by the study investigator.

^b^ Patient with scirrhous gastric cancer on day 113 that led to study drug discontinuation; considered not related to study drug.

^c^ Alcoholic liver disorder on day 183; considered not related to study drug (related to heavy use of alcohol) and did not interrupt study drug.

^d^ Patient had two serious adverse events (AEs; urinary tract infection [day 296] and hypoadrenalism [day 307]); both were considered not related to study drug, did not interrupt study drug, and resolved within 11 and 5 days, respectively.

^e^ Urinary calculus on day 384; considered not related to study drug (related to preexisting kidney calculus) and resolved by day 433.

^f^ Nonserious AE of inflammation, pain in extremity, and rash on day 1; considered not related to study drug.

^g^ Serious AE of ischemic heart failure leading to death on day 675; considered not related to study drug.

^h^ Nonserious elevation in liver enzymes; resolved 64 days later and did not lead to study drug disruption.

^i^ Nonserious aggravation of hepatic function disorder on day 85; considered not related to study drug and did not lead to study drug interruption; concomitant medications were discontinued.

^j^ Nonserious deterioration of hepatic function on day 86; considered not related to study drug and did not lead to study drug interruption.

^k^ Nonserious elevation in total bilirubin; resolved 176 days later and did not lead to study drug interruption.

## 4 DISCUSSION

Results from IMMspire demonstrated clinically meaningful efficacy with risankizumab, regardless of dose (75 or 150 mg); all patients achieved the primary end points of clinical response at week 16. Most patients achieved skin clearance assessed by PASI 90 at week 16. In addition, most of the patients who received risankizumab achieved DLQI 0/1 (no effect on patient's life) at week 16, which may suggest that risankizumab may help improve patient's quality of life. Overall, long‐term treatment with risankizumab demonstrated durable high rates of skin clearance in patients with GPP or EP. All patients who continued with risankizumab therapy maintained a clinical response at week 160.

Risankizumab was generally well tolerated by patients with GPP or EP, and no new safety signals were identified. The safety profile of risankizumab observed through 160 weeks of therapy and follow‐up through 180 weeks was consistent with the safety profile of risankizumab observed through 172 weeks of treatment in patients with psoriasis.^9^ In addition, safety findings are generally consistent with long‐term treatment with other biologics studied in Japanese patients with GPP or EP.^10^, ^11^

IMMspire has some limitations. First, the interpretation of efficacy and safety outcomes is limited by the lack of a placebo group. Second, the open‐label nature of the study may introduce bias as patients and investigators were aware of the study treatment. Last, the small sample size of the study may overestimate the magnitude of efficacy and AEs. Despite these limitations, findings from the present study suggest that there are positive treatment benefits for Japanese patients with GPP and EP.

In conclusion, the results from IMMspire showed clinically meaningful and durable efficacy of risankizumab over time in patients with GPP or EP. Efficacy was achieved at week 16 and maintained throughout treatment, and up to the last follow‐up visit. The safety profile was consistent with the safety profiles outlined in other psoriasis studies with risankizumab treatment. These results suggest that risankizumab may be an effective long‐term treatment option for GPP or EP.

## CONFLICT OF INTEREST

AbbVie participated in the study design; study research; collection, analysis, and interpretation of data; and writing, reviewing, and approving this manuscript for publication. All authors had access to the data, participated in the development and review of the document, and agreed in the decision to submit this manuscript for publication. No honoraria or payments were made for authorship. K.Y. has served as a clinical trials investigator for AbbVie, Boehringer Ingelheim, Eli Lilly Japan, LEO Pharma, Janssen, Maruho, MSD, Parexel, and UCB Japan. He has received research funding from AbbVie, Eisai, Eli Lilly Japan, Kyowa Kirin, Maruho, Sasaki Chemical, Sun Pharma, Taiho Yakuhin, and Torii Yakuhin. In addition, he has received speaker's fees and chair's fees from AbbVie, Astellas Seiyaku, Celgene, Daiichi‐Sankyo, Eisai, Eli Lilly Japan, Janssen, Kyowa‐Hakko Kirin, LEO Pharma, Maruho, Nippon Kayaku, Nippon Zouki, Novartis, Sun Pharma, Sato Seiyaku, Sanofi, Taiho Yakuhin, Tanabe Mitsubishi, and Torii Yakuhin. Y.O. has received grants from Eisai, Maruho, Shiseido, and Torii; consulting/advisory board fees from AbbVie, Amgen, Boehringer Ingelheim, Bristol‐Myers Squibb, Eli Lilly, Janssen, and Sun Pharma; speakers' bureau fees from AbbVie, Amgen, Boehringer Ingelheim, Bristol Myers Squibb, Celgene, Eisai, Eli Lilly, Janssen, Jimro, Kyowa Kirin, LEO Pharma, Maruho, Mitsubishi Tanabe, Novartis, Pfizer, Sanofi, Sun Pharma, Taiho, Torii, and UCB; and fees for participating in clinical trials sponsored by AbbVie, Amgen, Boehringer Ingelheim, Bristol‐Myers Squibb, Celgene, Eli Lilly, Janssen, LEO Pharma, Maruho, Pfizer, Sun Pharma, and UCB. I.Y., N.S., and I.M. are employees of AbbVie Inc. or AbbVie GK, and may hold AbbVie stock and/or stock options. A.M. has received honoraria from AbbVie, Ayumi Pharmaceutical, Boehringer Ingelheim, Celgene, Eisai, Eli Lilly, Inforward, Janssen, Kyowa Kirin, Maruho, Mitsubishi Tanabe, Nippon Kayaku, Novartis, Taiho, Torii, and Ushio. He has received research support from AbbVie, Eisai, Eli Lilly, Kyowa Hakko Kirin, LEO Pharma, Maruho, Mitsubishi Tanabe, Novartis, Taiho Pharma, and Torii. He has also served as a consultant for AbbVie, Boehringer Ingelheim, Bristol Myers Squibb, Celgene, Eli Lilly, GlaxoSmithKline, Janssen, Kyowa Kirin, Maruho, Mitsubishi Tanabe, Nichi‐Iko Pharmaceutical, Nippon Kayaku, Novartis, NPO Health Institute Research of Skin, Pfizer, Sun Pharma, Torii, and UCB.
